# Flavor and product messaging are the two most important drivers of electronic cigarette selection in a choice-based task

**DOI:** 10.1038/s41598-021-84332-4

**Published:** 2021-02-25

**Authors:** Allison N. Baker, Stephen J. Wilson, John E. Hayes

**Affiliations:** 1 Graduate Program in Neuroscience, The Pennsylvania State University, University Park, PA 16802 USA; 2 Sensory Evaluation Center, The Pennsylvania State University, University Park, PA 16802 USA; 3 Department of Psychology, College of the Liberal Arts, The Pennsylvania State University, University Park, PA 16802 USA; 4 Department of Food Science, College of Agricultural Sciences, The Pennsylvania State University, 220 Food Science Building, University Park, PA 16802 USA

**Keywords:** Olfactory system, Reward, Preclinical research, Translational research

## Abstract

Electronic cigarette use—vaping—is increasingly popular. Various product factors may influence an individual’s choice of e-cigarette. To provide an evidence base for e-cigarette regulation, a better understanding of the role different product attributes play in product preferences is needed. Here, we used conjoint analysis to quantify different factors that influence e-cigarettes choices, including flavors, nicotine level, customizability, or use of e-cigarettes to manage appetite/food craving. Young adults completed a set of choice-based conjoint tasks online. Choice Based Conjoint analysis (CBC) was used to determine utility scores for each attribute. Young adults (n = 587) who vaped at least once per week were included in analyses; gender differences were explored. Flavor was the most important attribute (48.1%), followed by product messaging (21.0%) and nicotine level (15.3%). Within flavor, confectionery and fruit flavors had the highest utility scores, while classic menthol and tobacco flavors had the lowest. Men and women differed in flavors, nicotine levels, and product messaging that appealed most. Among young adults who vape weekly, flavor is the most important factor in e-cigarette preferences. Gender also factors into e-cigarette preferences, especially for preferred nicotine level. Understanding why individuals choose particular e-cigarette products will help inform public health efforts and policy making.

## Introduction

Electronic cigarette (“eCig”) use, or “vaping,” is rapidly growing in popularity, especially among adolescents and young adults. Recent data indicate the prevalence of vaping among United States high school seniors (in the last 30 days) has more than doubled from 11.0% in 2017 to 25.9% in 2019^1^. While federal regulations in the United States (US) prevent combustible tobacco cigarettes from containing added flavors other than menthol, the regulations on eCig flavorings are less strict. As of early 2020, pod based eCig manufacturers must get premarket authorization from the Food and Drug Administration (FDA) before selling flavored eCig pods^2^. As of Fall 2020, no manufacturer has obtained such clearance; critically however, these rules do not apply to disposable eCigs. Thus, eCig users still have wide range of flavor profiles they can purchase: varieties such as fruits, desserts, cocktails, menthols, and tobacco are widely available, with thousands of options to meet a vast range of preferences. Taste and flavor are clearly key facets of electronic cigarette appeal^3,4^. While vaping might be less risky than smoking combustible cigarettes for those who already smoke, vaping also presents a substantial risk for initiation of nicotine use and dependence in those who would not otherwise smoke^5,6^. Accordingly, it is critical to develop a better understanding of the role of flavor in the preferences of eCigs to provide an evidence base for policy making and evidence-based regulation of these devices.

Prior work has explored why eCig users select flavored e-liquids. Soule and colleagues used concept mapping to explore reasons for use of flavored liquids among eCig users^7^. They identified five clusters of reasons for flavored e-liquid use, including Increased Satisfaction/Enjoyment, Better Feel/Taste than Cigarettes, Variety/Customization, Food Craving Suppression, and Social Impacts. Participants rated the degree to which several statements were reasons for their eCig use. Significantly higher ratings were observed for statements in the Increased Satisfaction/Enjoyment and Better Feel/Taste than Cigarettes clusters compared with statements from other clusters. Participants in their study also suggested added flavors could increase the rewarding effects of eCigs.

Consistent with this, results from multiple studies indicate that flavor is a major facet of eCig liking and use. Kim and colleagues assessed liking and disliking of several eCig flavors, finding that sweet flavors were preferred^8^. Litt, Duffy, and Oncken found eCig vaping rates were significantly influenced by flavor: tobacco and cherry flavors had the highest vaping rates, while chocolate flavors had the lowest rates^9^. In a systematic review of consumer preferences for eCig attributes, Zare and colleagues observed that adolescents, young adults, and older adults were all interested in non-tobacco flavors, especially Candy, Fruit, and Sweet flavors^10^. These studies all support the idea that flavor plays a key role in eCig preferences.

For this project, we used choice based conjoint analysis to quantify how various factors influence eCig preferences, and to determine the relative importance of each of these factors. Conjoint analysis is a decades old method used to systematically quantify preferences for specific product attributes. Critically, rather than directly asking participants to explicitly declare what factors they find most important when choosing a product (i.e., a stated preference), conjoint analysis asks participants to respond *holistically* to a series of product profiles, thereby revealing their preferences^11,12^. By systematically permuting product attributes in the profile of each product in the choice set, the relative importance of each can be determined quantitatively. Further, conjoint analysis is thought to increase ecological validity, as it does not require participants to explicitly identify or even be aware of reasons that underlie their specific preferences. That is, this method is able to reveal hidden or unconscious motivations without social desirability biases and post hoc reconstruction that may arise with traditional qualitative methods like structured interviews or focus groups. Here, we used choice-based conjoint (CBC) analysis, a variant where participants are shown multiple, competing product profiles simultaneously and are asked which they would choose from the set, similar to other work^13^. In a typical conjoint study, participants complete 12–30 choice tasks, with varied attributes presented in each. Researchers can then deduce which features are most desired in the product and which attributes have the greatest influence on participant choice. This is a more realistic trade-off scenario, as opposed to explicitly asking participants to state what aspects they prefer or what is important to them.

The objective of this study was to investigate factors that drive acceptability of eCigs in young adults, and to quantify their relative importance. These factors include nicotine level, flavors, device style, customizability, and use of eCigs for weight/appetite control or as a means of managing food craving. Classically, conjoint analysis is primarily exploratory and descriptive, so we did not define specific hypotheses to be tested. That said, we did expect a priori to see certain patterns in our data. First, we expected nicotine level and flavor would be among the most important attributes. Second, we expected that appetite control and satisfaction of food cravings would be important factors in eCig use, especially in women. Contrary to this prediction, however, conjoint analysis revealed that control of appetite and cravings, and device style, were not meaningful drivers of choices in this cohort. Accordingly, the manuscript focuses primarily on the attributes with the highest utility scores.

## Methods

### Participants

Participants between 18 and 30 years of age were recruited by a third-party market research agency (Dynata LLC, formerly Survey Sampling International, based in Shelton, CT). To qualify, individuals had to be living in the United States and must have used an electronic cigarette (eCig) at least twice within the past 12 months. All data were collected in February 2019 (i.e., before the nicotine purchase age in the US was raised to 21 years in December 2019, and before Juul and Vuse stopped selling flavored pods in early 2020). All participants provided informed consent and could exit the survey at any time. Qualified participants received a link to the questionnaire, comprised of several sections. All study procedures complied with all relevant guidelines, and they were approved by the Institutional Review Board at the Pennsylvania State University (STUDY00010047).

### Conjoint analysis

Briefly, in conjoint analysis, specific product elements are grouped into categories (sometimes called attributes or silos), and each category is composed of multiple elements that can be nominal or ordinal elements (i.e., levels). Individual elements from each category are permuted via a factorial experimental design to create unique product concepts or profiles. In a CBC task, participants are shown multiple product profiles and are asked to select their preferred product. Here, each CBC task had 3 profiles to pick from, and a fourth ‘none of these’ option was also provided, consistent with recommended best practices. Each profile only contained 3 attributes, and as shown in an example screenshot (Supplemental Fig. 1), profiles were words only; photographs or images were not included. Each participant completed a total of 24 CBC tasks. All possible permutations were presented across the entire cohort, but each participant did not see every possible concept (i.e., an incomplete block design). The product elements tested here were chosen based on our previous experience with conjoint analysis, informal review of vaping websites, and prior literature (e.g.^14,15^). The device styles were "Compact, looks like a cigarette (round, long design with glowing tip)," "Compact, does not look like a cigarette (boxy, flat design)," "Cylindrical e-cig rig with a tank that can be filled with your choice of e-liquid," and "Boxy e-cig rig with an LED screen and a tank that can be filled with your choice of e-liquid." The nicotine levels were 0, 3, 6, 12, and 18 mg/ml. The flavors assessed here were Classic Tobacco, Cuban Cigar, Cucumber Mint, Menthol, Cherry, Mango, Birthday Cake, and Apple Pie. The options for product messaging were "Satisfies food cravings," "Reduces stress," "Can produce big vapor clouds," "Suppresses appetite," "Relaxes you," and "Helps you focus." For the purchase availability attribute, the options were "Available online," "Available in convenience stores," and "Available in smoke shops." Finally, in the health warning category, the levels were "This product contains nicotine, a chemical known by the state of California to cause birth defects or other reproductive harm," "This product contains nicotine, an addictive chemical," and "Not for sale to minors." Data were collected using Discover-CBC from Sawtooth Software (Orem, UT), a specialized conjoint analysis software platform; participants were recruited by Dynata and they completed the survey using their own devices. Utility functions for attributes (i.e., relative importance), and segmentation were performed within Discover (details below).

**Figure 1:**
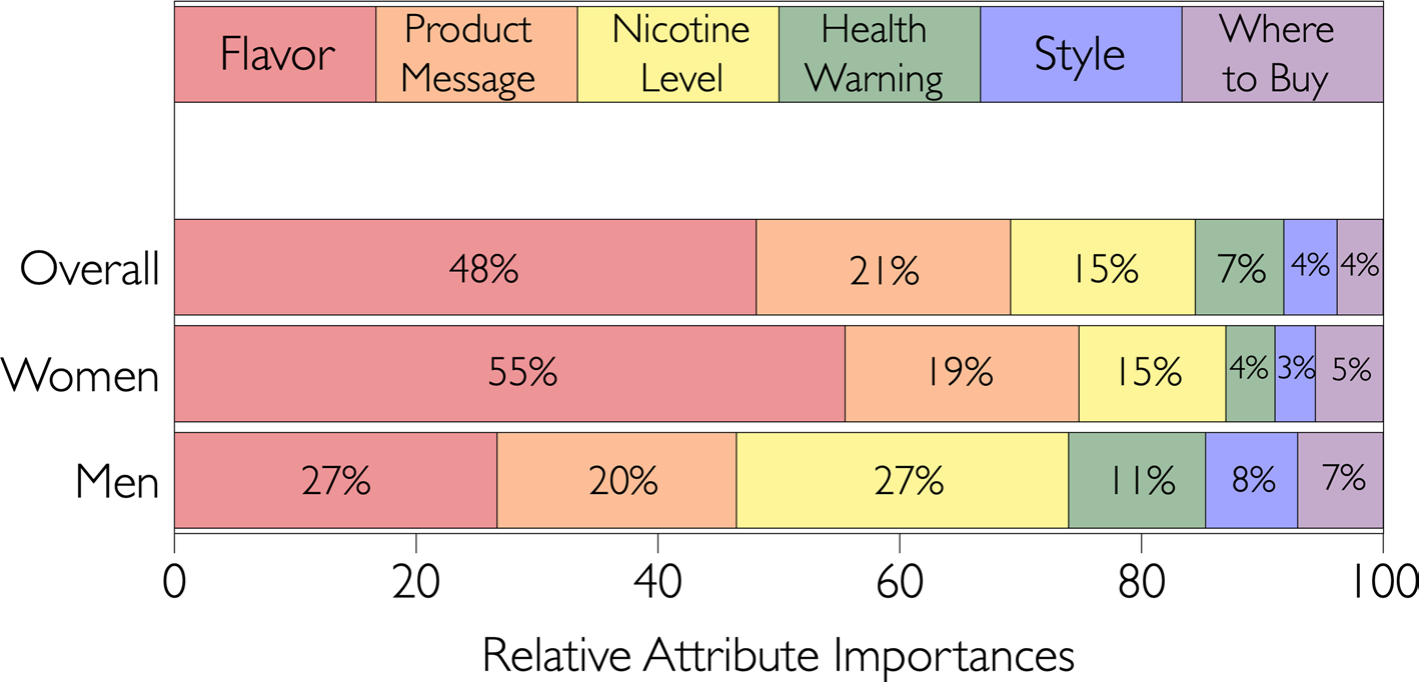
Relative importance of various attribute categories, based on discrete choices, for all participants (n = 587), and for women and men separately.

### Demographic and nicotine use information

After evaluating 24 CBC tasks, participants were asked to provide their age, self-reported ethnicity, gender, whether or not they were actively dieting, how frequently they vape/use an eCig, whether or not they purchase eCigs themselves, and where they purchase eCigs. Participants then completed the Penn State Electronic Cigarette Dependence Index (PSECDI), a dependence questionnaire tailored toward eCig use^16^. Finally, they answered demographic questions and answered a subset of questions from the Wisconsin Inventory of Smoking Dependence Motives (WISDM-68) that we modified to ask about eCigs rather than combustible tobacco cigarettes^17^.

### Analysis

Participants with incomplete data were excluded (details below). All CBC data were analyzed using vendor-provided tools in the conjoint software platform, Discover-CBC. Specifically, individual-level logit with Hierarchical Bayes estimation was used to determine utility values (relative importance) of individual elements, including flavor options, nicotine level, device style, product claims, purchase availability, and health consequences. The full WISDM-68 includes 68 questions that form 13 subscales identified by the original authors. Here, to reduce participant burden, we only collected 6 subscales, specifically: Cognitive Enhancement, Craving, Cue Exposure-Associative Processes, Social-Environmental Goads, Taste and Sensory Processes, and Weight Control. Subscale scores were calculated by taking the mean of the items comprising each subscale. For the PSECDI, the sum of participants’ answers across items was taken. Raw dependence scores were calculated according to the questionnaire scoring method^16^.

## Results

### Participant characteristics

Based on initial recruitment criteria, 1,472 participants qualified for the online survey, but complete data was only available from 620 individuals. Most completers reported vaping at least once per week, with 4.9% using eCigs less than once per week. Individuals who vaped less than once per week were excluded from further analyses, resulting in a final n of 587, that was 64.8% white and 47.5% male. Additional demographics are provided in Table 1. The average PSECDI score across all participants was 8 ± 2.83, which falls in the low dependence category^16^. The means for the various WISDM subscales are shown in Table 1. These means are roughly similar to those reported elsewhere for light and heavy daily smokers^18^, except for the weight control subscale, which was roughly 1 point higher. Still, this apparent difference was not reflected in the conjoint data reported below.

**Table 1:** Demographics of young adult (18–30 years) participants who vape at least once a week.

Category	Overall		Men		Women	
	Sample (n = 587)	Prevalence (%)	Sample (n = 279)	Prevalence (47.5%)	Sample (n = 308)	Prevalence (52.5%)
**Average age**	26	–	26	–	26	–
**Ethnicity**						
American Indian or Alaska Native	11	1.9	5	1.8	6	1.9
Black or African American	91	15.5	42	15.1	49	15.9
Hispanic or Latino	74	12.6	35	12.5	39	12.7
White	378	64.4	180	64.5	198	64.3
Asian/Pacific Islander	33	5.6	17	6.1	16	5.2
**Actively Dieting**	285	48.6	148	53.0	137	44.5
**Vape Frequency**						
Multiple times per day	293	49.9	138	49.5	155	50.3
Once per day	115	19.6	59	21.1	56	18.2
5–6 times per week	113	19.3	56	20.1	57	18.5
2–4 times per week	52	8.9	21	7.5	31	10.1
Once per week	14	2.4	5	1.8	9	2.9
**Purchase yourself**	543	92.5	257	92.1	286	92.9
**Where purchase**						
Online	199	33.9	107	38.3	92	29.9
Smoke shop	296	50.4	124	44.4	172	55.8
Convenience store	86	14.7	46	16.5	40	13.0
Other	6	1.0	2	0.7	4	1.3
**Average PSECDI score**	8 ± 2.83	–	8 ± 2.88	–	8 ± 2.77	–
**WISDM subscale averages**						
Cue exposure	4.69 ± 1.41	–	4.95 ± 1.28	–	4.45 ± 1.47	–
Craving	4.69 ± 1.52	–	4.93 ± 1.39	–	4.48 ± 1.61	–
Cognitive enhancement	4.86 ± 1.5	–	5.16 ± 1.36	–	4.58 ± 1.57	–
Social environmental goads	4.70 ± 1.61	–	4.99 ± 1.46	–	4.43 ± 1.69	–
Taste and sensory processes	5.28 ± 1.29	–	5.35 ± 1.26	–	5.23 ± 1.31	–
Weight control	4.48 ± 1.64	–	4.84 ± 1.54	–	4.16 ± 1.67	–

### *Top line results across the entire cohort (n* = *587)*

**Figure 2:**
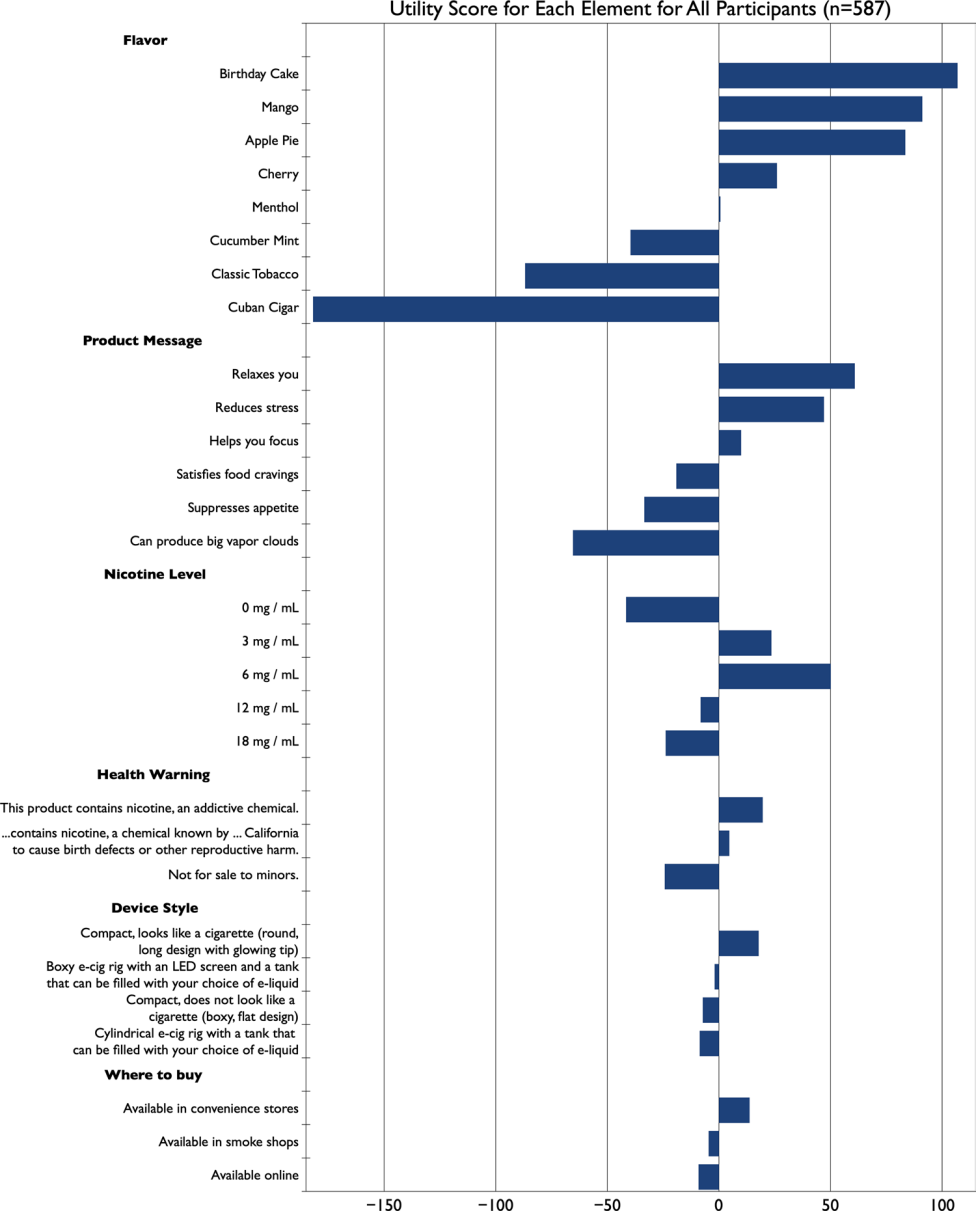
Utility scores of individual elements across all participants, ordered vertically by importance of each category. Elements with positive scores are to the right, and negative scores are to the left.

Overall, flavor was the most important attribute when choosing an eCig product, as shown in Fig. 1. Utility scores for individual flavors are depicted in Fig. 2. A larger, positive utility score indicates that specific element had a larger, positive impact on preference. Likewise, a larger, negative utility score indicates that specific element had a larger, negative impact on preference. Confectionery (Apple Pie, Birthday Cake) and fruit (Cherry, Mango) flavors had the highest utility scores, whereas classic menthol and tobacco flavors were the lowest. Notably, the utility score for Menthol was close to zero, and Cucumber Mint, Cuban Cigar, and Classic Tobacco all had negative utility scores for this cohort of young adults.

Relative utility scores for product messaging deviated substantially from our original hypotheses. We had expected appetite suppression and satisfaction of food cravings would be major drivers of product selection. However, as shown in Fig. 2, these were among the lowest utility scores, along with “produces big vapor clouds.” Conversely, “relaxes you” and “reduces stress” had the highest utility scores, which is generally consistent with data from the nicotine level and health warning categories that are discussed below. Collectively, these data suggest user beliefs about expected outcomes (accurate or not) play a major role in electronic cigarette desirability, consistent with prior work on other tobacco products.

Contrary to expectations, the overall importance of nicotine level across the entire cohort was surprisingly small, at least relative to flavor (see Fig. 1). Relative utility scores for individual nicotine levels are shown in Fig. 2. Notably, the strongly negative score for 0 mg/ml nicotine indicates that, as a whole, the young adults studied here do not vape simply for flavor, and some amount of nicotine is desired. Notably, the highest nicotine levels also show negative utility scores, presumably due to harshness that occurs with high amounts of nicotine (e.g.,^8,19,20^).

Results from the health warning category are consistent with data above for 0 mg/ml products. That is, as Fig. 2 shows, the utility score for the health warning “This product contains nicotine, an addictive chemical” has a positive utility score. This suggests young adults who vape regularly want nicotine in the products they choose. While intuitive, a positive utility score of a message nominally intended as a health warning is a critical insight—it implies that a well-meaning message intended to inform buyers of the deleterious health impact of eCigs is actually viewed as a positive product attribute. Conversely, the “Not for sale to minors” warning has a negative utility score, despite our sample consisting of individuals aged 18 to 30.

Regarding device style, the magnitude of utility scores was relatively small in either direction. We had expected the highest utility scores for “Compact, does not look like a cigarette (boxy, flat design)”—that is, devices like a Juul. Instead, the option “Compact, looks like a cigarette (round, long design with glowing tip)” had the highest score (+ 17.8 vs. − 7.2 for flat boxy designs). The other two device styles also had negative utility scores (see Fig. 2). Still, the overall attribute importance for device style is quite small (4.4% in Fig. 1) in 18-to-30-year old adults and should not be overinterpreted. Whether device style may be more important in other age groups remains to be determined.

Last, across all 587 young adults, the least important driver of product selection was purchase location (see Fig. 1). As might be expected, the relative utility scores for these individual elements were quite small (see Fig. 2), although the scores did go in opposite directions: “Available in convenience stores” was positive (+ 13.75), while “Available in smoke shops” (− 4.62) and “Available online” (− 9.13) were negative.

### Segmentation of participants by gender

Since we originally hypothesized women would give higher utility scores to products that may facilitate weight control, we separated the cohort by gender and re-ran the logit model in the Sawtooth Discover platform. We focused on the top three product attributes: flavor, product messaging, and nicotine level (see Fig. 1). In the overall cohort, flavor was most important (48.1%), followed by product messaging (21.0%), then nicotine level (15.3%); for the women in our sample, these attributes' importances were rated in the same order: flavor (55.5%), messaging (19.4%), and nicotine level (12.2%). Interestingly, men rated nicotine level and flavor as roughly equally important (27.5% vs. 26.7%, respectively), with product messaging being third (19.8%). These data suggest women and men may have different motivations for selecting eCig products.

Women and men were fairly similar in their flavor preferences, though a few key differences became apparent. Women found specific flavors more polarizing, with utility scores ranging from -216.24 for Cuban Cigar to + 116.62 for Birthday Cake; conversely, men did not feel as strongly about flavor, with utility scores ranging from − 97.16 for Cuban Cigar to + 62.95 for Birthday Cake (see Supplemental Fig. 2, left panel). Still, both men and women gave negative ratings to Cuban Cigar, Classic Tobacco, and Cucumber Mint; Menthol was slightly negatively rated by women and slightly positively rated by men. Cherry, Apple Pie, Mango, and Birthday Cake were all positively rated, and women rated the latter three as more positive than did men. Because the magnitude of women’s utility scores for flavor were larger than for men, it appears women are more motivated by flavor.

Next, we observed gender differences in product messaging. Both women and men were interested in the messaging “Relaxes you” and “Reduces stress,” though men valued the latter as more important than women (+ 53.86 vs. + 36.47). Men had a positive score (+ 44.26) for “Helps you focus,” while this was scored negatively by women (− 9.99). In our cohort, neither women nor men were interested in appetite suppression, satisfaction of food cravings, or production of large vapor clouds, as both groups gave negative utility scores to these product claims. These scores (shown in Supplement Fig. 2, right panel) suggest that particular product messaging may appeal more to women over men, or vice versa.

**Figure 3:**
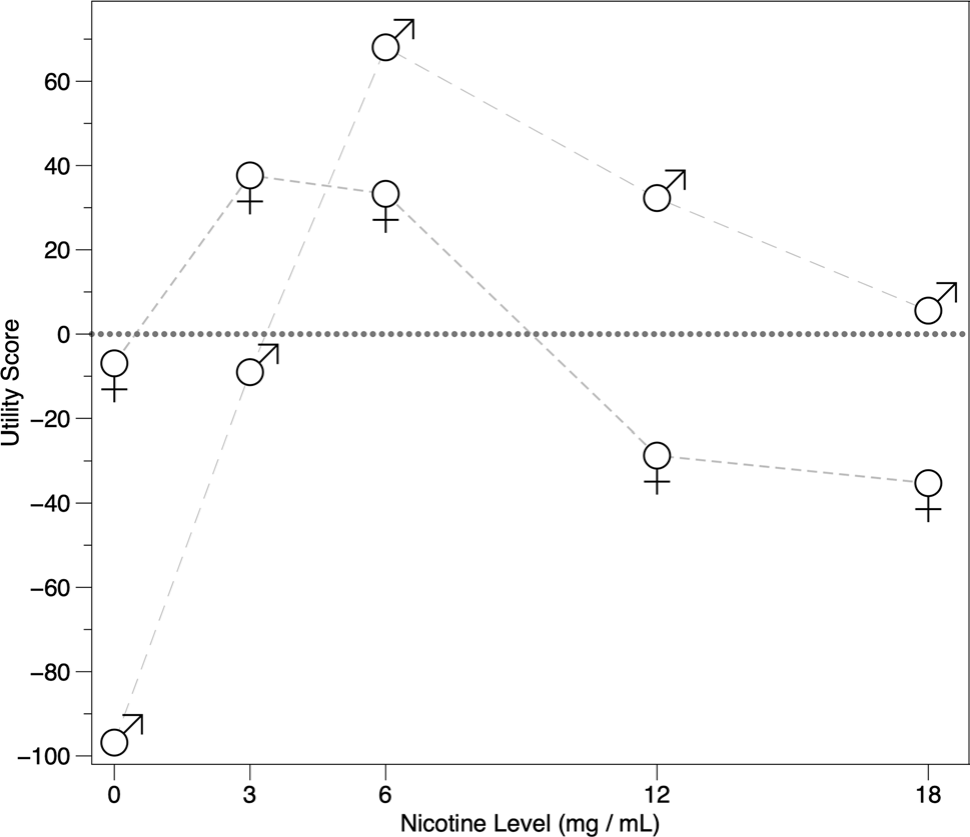
Utility scores for the five nicotine levels provided within the choice task. When split by gender, the preferred nicotine level for women was lower than the preferred level chosen by men. Elements with positive scores are shown above the dotted line, while negative scores fall below the dotted line.

We next examined differences in nicotine level utility scores by gender (Fig. 3). Both men and women had positive utility scores for 6 mg/ml nicotine (men + 68.05, women + 33.28). Interestingly, women also had a positive utility score for 3 mg/ml nicotine (+ 37.65), while this had a negative scores for the men (− 9.04); conversely, women had negative scores for 12 mg/ml and 18 mg/ml nicotine (− 28.73 and − 35.26, respectively), while men had positive scores (+ 32.28 for 12 mg/ml and + 5.56 for 18 mg/ml). For both, 0 mg/ml nicotine had negative scores, with men having a substantially lower score than women. These findings suggest women generally prefer lower nicotine levels (optimal of 3 to 6 mg/ml nicotine) when compared to men (optimal of 6 to 18 mg/ml nicotine).

For young adults studied here, men and women only had positive utility scores for the cig-a-like design (“Compact, looks like a cigarette (round, long design with glowing tip)”) while the other three device styles were scored negatively (boxy, flat compact design and both customizable eCig rigs). Men were interested in purchasing online or in convenience stores, but not in smoke shops; women were interested in purchasing in convenience stores or in smoke shops, but not online (not shown). Finally, the health warning 'Not for sale to minors' was rated negatively by both groups.

Taken together, segmenting our data by gender reveals differences in overall importance of eCig attributes in men and women, with varied preferences for specific product elements within those attributes.

## Discussion

Here, we used choice-based conjoint (CBC) analysis to determine the eCig attributes that are most important among young adults who regularly vape. Choice based experiments have been used previously in the context of nicotine (e.g.,^13^)—here we looked at a U.S. based sample of adults who vape, in contrast to Czoli and colleagues, who studied a Canadian sample of smoking and non-smoking youths, young adults, and adults^13^. Also, we used different flavors in our study. Here, flavor was the most important attribute in the overall cohort, followed by product messaging and nicotine level. This is not surprising, as multiple studies have acknowledged the importance of flavor^8,10,21,22^. When split by gender, women rated flavor as more important than did men, followed by product messaging and nicotine level, whereas men gave roughly equal weight to nicotine level and flavor, followed by product messaging.

Across the entire cohort, sweet flavors (fruit, confectionery) that were least similar to traditional combustible tobacco cigarette flavors had the highest utility scores. This recapitulates previous findings that sweet flavors are highly preferred^8,10^. While we selected only two flavor options for each category, there were thousands of flavors available on the U.S. market when the study was conducted. While we did observe some gender differences in magnitude of scores, all four sweet flavors tested here received positive scores in both women and men. As of 2020, the only pod-based flavors available in the U.S. market are tobacco and menthol flavors; however, diverse flavors are still available in disposable eCigs (e.g., PuffBar). Also, the cohort tested here was ethnically and racially diverse, but we were not powered to look for differential flavor preferences across race and ethnicity. Given the historic targeting of specific flavors to certain groups by the tobacco industry (e.g., marketing of menthol cigarettes to African Americans), future work should explore whether such differences may exist for eCig flavors.

We initially hypothesized appetite suppression and satisfaction of food cravings would be potentially appealing product claims, as many studies have reported that nicotine may be used as a weight loss or weight control method (e.g.,^14,23,24^). While we were broadly interested in emerging adults, young women may present a particularly vulnerable population, as they are also likely to have concerns about weight/body image and may first try nicotine products around this age^25^. Prior studies have investigated the effect of eCigs on weight^15^, but this had not been specifically explored in young women, or within the context of flavor when our study was designed and executed. Subsequently, Morean and colleagues tested a school-based sample of 13 to 19 olds, finding that 13.8% of adolescents reported vaping flavored e-liquids for appetite control and 9.3% for weight loss^14^. Additionally, using flavored e-liquids for either of these purposes was associated with more frequent vaping, as well as using more flavored e-liquids. In particular, candy-flavored e-liquids were associated with vaping for appetite control. When designing our study, we had hypothesized that above and beyond other reasons for their use, eCigs may appeal to young women (18 to 30 year olds) as a weight control method. Specifically, enjoyable flavors may help indirectly control weight by satisfying food cravings, and nicotine can directly control weight by reducing appetite. However, based on revealed preferences from a choice-based task, it is clear that weight control was *not* an important factor in eCig choices in our cohort, despite stated importance in other studies. Further, our WISDM weight control subscale mean was higher than in other samples, but this was not reflected in the actual choices participants made here. Whether this would also be true in other younger cohorts is unknown. Whether the discrepancy between our results and those of Morean and coworkers is due to differences in age (13 to 19 vs. 18 to 30 years), sample (national vs. Connecticut), or method (survey vs. choice-based experiment) is unclear and warrants more research.

We observed some inconsistency between declared interest via self-report and revealed importance via choice-based utility scores for purchase location. When questioned, most participants said they purchase online or at a smoke shop, yet utility scores for these locations were negative, and the convenience store score was positive. These discrepancies highlight a potential advantage of conjoint analysis, as it can reveal preferences that may not be apparent in self-report measures. Still, additional work is warranted to confirm these findings and resolve these discrepancies.

Contrary to expectations, nicotine level did not appear to be as important a factor in the overall analysis as originally anticipated. It may be that individuals can self-regulate their nicotine intake, so if the available nicotine level is higher or lower than desired, they can alter their vaping topography to achieve the desired effect. Other studies have delved into the importance of nicotine level and preferences for different strengths (see^10^), though it is possible that stated preferences, once again, differ from revealed preferences in a choice-based task. Notably, nicotine level was the most important attribute for men, and third most important for women, suggesting gender differences in salience of nicotine level. This is consistent with evidence that smoking may be driven to a greater extent by nicotine in men versus women (e.g.,^26–28^).

The message “Produces big vapor clouds” had negative utility scores in all groups. We anticipated those who vape as a hobby may enjoy cloud production, though it may be the case that there are differing motivations for vaping, and the cohort studied here was more focused on flavor. Based on online media (e.g., YouTube, Reddit, TikTok), there is a common stereotype that eCig users are interested in large vapor clouds and building custom rigs. Studies that leverage social media (e.g.,^29^) or recruit from eCig messaging boards may oversample members of this subpopulation. Here, we did not observe any evidence of this, suggesting our cohort may better align with the general public rather than internet driven stereotypes of those who vape.

Other gender differences were also apparent. Women clearly preferred confectionery and fruit flavors and had negative utility scores for the more 'traditional' flavors: Classic Tobacco, Cuban Cigar, Cucumber Mint, and Menthol. They were interested in 3 to 6 mg/ml nicotine, but not 0, 12, or 18 mg/ml nicotine. Product messaging that appealed to women included “Reduces stress” and “Relaxes you.” Men also preferred confectionery and fruit flavors and had negative scores for most 'traditional' flavors, with the exception of Menthol; however, overall the magnitude of men's flavor utility scores were smaller than for women. Men were interested in 6 to 18 mg/ml nicotine, but not 0 or 3 mg/ml nicotine. Product messaging that appealed to men included “Reduces stress,” “Relaxes you,” and “Helps you focus.” We observed minor differences in scores for device style, purchase availability, and health warnings, though these attributes were the least important for both groups. Collectively, these findings indicate men and women differ not only in the attributes that are most important to them, but also in which specific product elements are most appealing.

Limitations of the present study should be mentioned. Here, we tested individuals aged 18–30: some effects may generalize broadly, but we anticipate that underage teens may have substantially different priorities in eCig selection than for the of age adults studied here. Originally, we planned to recruit underage individuals, but ultimately demurred due to ethical concerns. Still, eCigs seem to have great appeal to adolescents, especially in terms of concealability and sweet flavors^10^. Also, we only asked about vaping, not use of combustible tobacco cigarettes or other nicotine products, so it is possible that individuals studied here were dual users. We might expect to see differences in flavor and product preferences in exclusive eCig users versus eCig/combustible cigarette dual users. Here, we used "Available in smoke shops" as a purchase location rather than"Available in vape shops." While many smoke shops carry eCig products along with hookah and other combustible tobacco items, we cannot rule out a bias in responses due to this wording choice. Additionally, we do not have information on how long each participant has been an eCig user: this may be salient not only in terms of level of nicotine dependence but also in terms of the dominant device on the market at the time of eCig initiation (e.g., Blu versus Juul versus PuffBar). More experienced eCig users or former smokers may have different preferences than those who recently took up vaping. Finally, due to an oversight, no attempt was made to assess past or concurrent use of combustible cigarettes or other nicotine containing products, a factor that should be included in future studies.

In conclusion, in a study of revealed preferences using choice based conjoint analysis, flavor was the most important factor in eCig appeal in a cohort of 18-to-30 year old adults in the U.S., followed by product messaging and nicotine level. We also found evidence men and women differed in what elements were most appealing. Understanding why individuals choose particular eCig products should help inform public health efforts and policy making.

## Supplementary Information

Supplementary Information 1.

## Data Availability

The datasets generated during and/or analysed during the current study are available from the corresponding author on reasonable request.
