# Supplementary material for: Flavor and product messaging are the two most important drivers of electronic cigarette selection in a choice-based task
Source: Sci Rep. 2021 Feb 25;11:4689. doi: 10.1038/s41598-021-84332-4 (PMC7907091; doi:10.1038/s41598-021-84332-4)
Supplement: Supplementary file 1 — Supplementary Information 1. [file 41598_2021_84332_MOESM1_ESM.docx]

**Supplementary Materials for:**

**“Flavor and product messaging are the two most important drivers of
 electronic cigarette selection in a choice-based task”**

Allison N. Baker^1,2^, Stephen J. Wilson^3^, and John E. Hayes^2,4,*^

^1^Graduate Program in Neuroscience,

^2^Sensory Evaluation Center,

^3^Department of Psychology, College of the Liberal Arts,

^4^Department of Food Science, College of Agricultural Sciences,

The Pennsylvania State University, University Park PA 16802

*Corresponding Author:

Dr. John E. Hayes

Department of Food Science

Pennsylvania State University

220 Food Science Building

University Park, PA 16802

814-863-7129 (voice)

[jeh40@psu.edu](mailto:jeh40@psu.edu)

Twitter: @TasteProf

Running Title: Drivers of eCig selection in adults who vape regularly


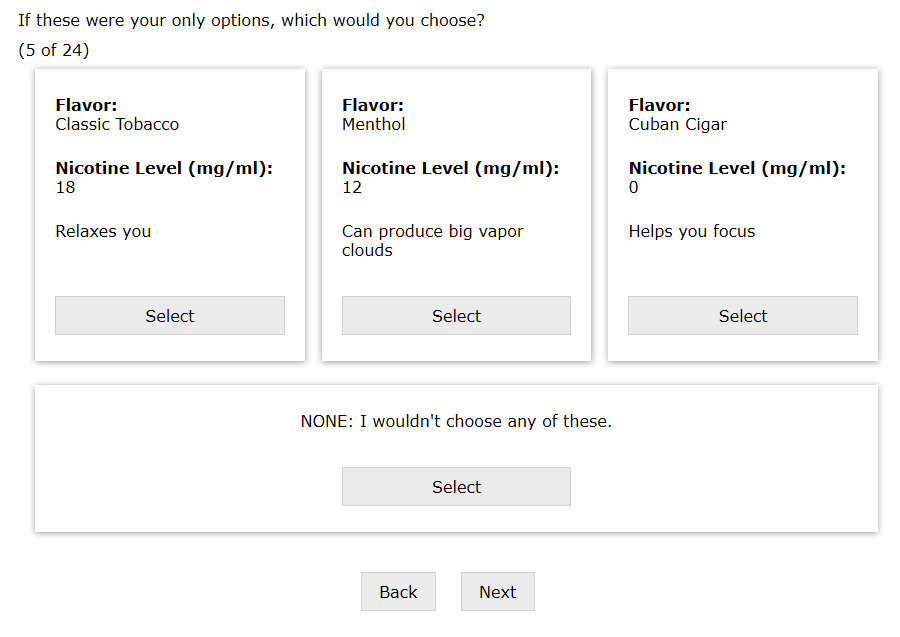


**Supplemental Figure 1: Screenshot of a single choice-based conjoint task presented via the Discover-CBC platform. Each study participant completed a total of 24 of these tasks.**


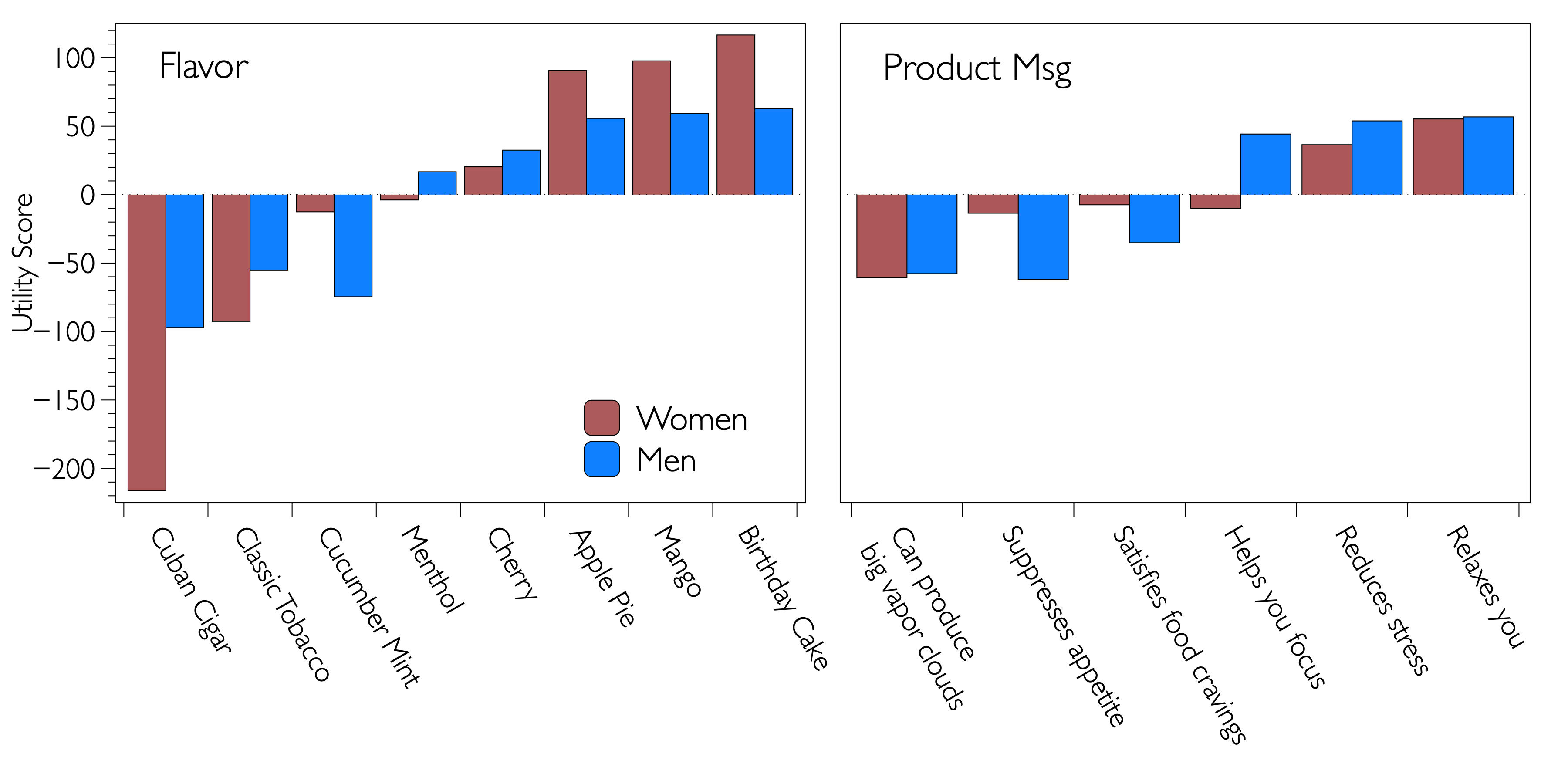


**Supplemental Figure 2: Utility scores of top 2 attributes (flavor & product messaging), split by gender.**
